# Clinical relevance of the reappraisal of negative hormone receptor expression in breast cancer

**DOI:** 10.1186/2193-1801-2-375

**Published:** 2013-08-09

**Authors:** António E Pinto, Filipa Areia, Teresa Pereira, Paula Cardoso, Mariana Aparício, Giovani L Silva, Mónica C Ferreira, Saudade André

**Affiliations:** Serviço de Anatomia Patológica, Instituto Português de Oncologia de Lisboa Francisco Gentil, E.P.E.R. Prof. Lima Basto, 1099-023 Lisboa, Portugal; Centro de Estatística e Aplicações da Universidade de Lisboa, Lisboa, Portugal; Departamento de Matemática do Instituto Superior Técnico da Universidade Técnica de Lisboa, Lisboa, Portugal

**Keywords:** Breast cancer, Estrogen receptors, Progesterone receptors, Immunohistochemistry, Prognosis

## Abstract

**Background:**

Accurate assessment of estrogen (ER) and progesterone (PR) receptors is critical in predicting the response to endocrine therapies in breast cancer.

**Material and methods:**

From a series of 360 patients with breast invasive carcinoma assessed for hormone receptors by immunohistochemistry (IHC) in the 90’s, we re-analysed, on the same tumour material, the cases considered negative (n = 164), i.e., ER-/PR- (n = 95), ER+/PR- (n = 63) and ER-/PR+ (n=6), and 16 of 196 ER+/PR+ tumours with unfavourable outcome. Concordance between the previous IHC (Streptavidin-Biotin-Peroxidase) method and the current one (Peroxidase-Indirect-Polymer) was determined by the McNemar’s test. Relapse-free (RFS) and overall survival (OS) were estimated by the Kaplan-Meier method.

**Results:**

From 101 ER- and 158 PR- cases, 38 (37.6%) and 58 (36.7%) became positive, increasing ER and PR expression from 71.9% and 56.1% to 82.5% and 72.2%, respectively (P<0.001). All 16 ER+/PR+ cases maintained their co-positivity, while all ER-/PR+ tumours changed to ER positive. Kaplan-Meier survival curves showed significant differences related to RFS and OS for PR, either in the whole series or in the subset (n = 151) submitted to hormonal treatment. The patients’ subgroup with ER+/PR- tumours exhibited the worst prognosis.

**Conclusion:**

The current IHC method improves the clinical usefulness of ER/PR assessment by decreasing the rate of false negative results.

## Introduction

The assessment of estrogen (ER) and progesterone (PR) receptors is predictive of the response to endocrine therapeutic strategies in breast cancer (Hammond et al. 2010; Allred et al. 2009; Goldhirsch et al. 2009). Along with this fundamental ability, the biomarkers analysis has shown, mainly for PR, prognostic significance as well (Pinto et al. 2003; Pinto et al. 2013). Therefore, an accurate determination of hormone receptor expression is critical in the management of breast cancer patients, both in the adjuvant and metastatic settings (Diaz & Sneige 2005; Elledge et al. 2000).

Currently, the most widely used technique for assessment of ER and PR status is immunohistochemistry (IHC) on formalin-fixed paraffin-embedded material (Allred et al. 2009). Its main advantages over other techniques (e.g., ligand binding assays or RT-PCR) stem from the easy, safe and relatively inexpensive application in routine practice, together with the possibility of morphological evaluation of small specimens and discrimination between benign and malignant cells (Hammond et al. 2010; Harvey et al. 1999).

A wide range of variability factors can affect, however, IHC methodology (Hammond et al. 2010; Bartlett et al. 2011), from pre-analytical variables, such as type of fixative and time of fixation, until scoring methods (and thresholds for positivity) for interpretation of tumour nuclear immunostaining slides. Other potential sources of analytical discordance include the choice of antibodies, antigen retrieval techniques, detection systems and quality control. Recently, increased attention has been paid on two controversial issues with clinical relevance: the high rate of false negative results (Hammond et al. 2010; Hede 2008; Allred 2008; Viale et al. 2007; Fisher et al. 2005), and the establishment of different cut-off points for distinguishing positive from negative biomarkers expression (Fisher et al. 2005; Regan et al. 2006; Cheang et al. 2006; Dowsett et al. 2008). It is obvious that both problems have direct impact on the important decision making of selecting patients for adjuvant hormonal therapies in breast cancer.

In order to compare the hormone receptor expression assessed by two IHC methods distant in time and investigate the prognostic implications of using various cut-off values to define ER and PR positivity, we decided to make the reappraisal of ER and PR status, using current IHC methodology, on the same previously analysed tumour samples of patients with breast cancer diagnosed in the 90’s in our Institution.

## Material and methods

### Clinico-pathological data

The series investigated encompassed 360 female patients with primary breast invasive ductal carcinoma, diagnosed and treated at Portuguese Oncology Institute (IPO) Lisbon Center, between August 1990 and November 1999. The inclusion criteria were the availability of ER and PR reporting data and complete follow-up information for patients. The study was carried out following guidelines approved by Comissão de Ética do IPOLFG the local institution ethical board. Patients had not been treated prior to surgery and none had metastatic disease at diagnosis. Their mean (and median) age was 59 years, ranging from 23 to 88 years. Most of them (n=253) were submitted to modified radical mastectomy and the remaining (n = 107) to breast conserving surgery. Adjuvant chemotherapy was given to 144 (40%) patients, while 151 (41.9%) received hormonal treatment. The histological type and pathological staging of breast carcinomas were evaluated according to WHO classification (Lakhani et al. 2012). Tumour differentiation was assessed using the Elston and Ellis grading system (Elston & Ellis 1991). Ploidy status was analysed by DNA flow cytometry. Table 1 shows, in detail, the clinico-pathological and ploidy characteristics of the series investigated.

**Table 1:** **Clinico**-**pathological features and DNA ploidy status of the series investigated (n=360)**

Variables	N (%)
Grade of differentiation	
G1	89 (24.7)
G2+G3	271 (75.3)
Tumour size	
pT1	157 (43.6)
pT2+pT3	203 (56.4)
Nodal status	
pN0	203 (56.4)
pN1	157 (43.6)
DNA ploidy	
Diploid	145 (40.3)
Aneuploid	215 (59.7)

*N* Number of patients.

Follow-up information was obtained by review of the patients clinical records. Outcome measures investigated were the relapse-free survival (RFS), that is, the time elapsed between diagnosis and the date of first local or distant recurrence, and the overall survival (OS), which is defined as the interval between diagnosis and death from the disease. Patients not experiencing the relevant end point were censored at last clinical observation.

### Hormone receptor expression

Table 2 summarizes the technical steps followed in the two IHC methods for assessing ER and PR expression. The IHC method used in the 90’s was performed on tumour paraffin-embedded material according to the streptavidin-biotin complex peroxidase technique (Hsu et al. 1981). The results were recorded as the percentage of positively stained target cells, positivity being defined as samples with more than 10% stained neoplastic cell nuclei. The intensity of staining was not evaluated.

**Table 2:** **Technical protocols used by the two IHC methods**

Technical steps		IHC methods
	Streptavidin-Biotin-Peroxidase	Peroxidase-Indirect-Polymer
	(90’s)	(currently)
Endogenous peroxidase blocking	2% H_2_O_2_	3% H_2_O_2_
Antigenic retrieval	Citrate buffer pH 6.0 in pressure cooker, 6´ in highest pressure	CC1 buffer pH 9.0, 52´
Primary monoclonal antibodies	NCL-ER-6F11 / NCL-PGR (Novocastra), diluted 1:10, 30´, room temperature	ER Ventana 790–4324 (SP1), pre-diluted, 60´, 37°C
		PGR Ventana 760–4296 (1E2), pre-diluted, 28´, 37°C
Secondary antibody	Biotinylated rabbit anti-mouse (E413, Dako), diluted 1:250, 30´	*Ultra*view universal DAB (760–500, Ventana), 8´, 37°C
Detection systems	StreptABC Complex (K0377, Dako), diluted 1:100, 30´, room temperature	
Controls	Negative: Primary antibody omission	Breast carcinoma tissue microarray (TMA) including “negative tumour, with normal glandular epithelium, positive tumour with moderate expression (30-70%), and positive tumour with high expression (≈ 100%)”
	Positive: Breast carcinoma positive case	

All cases considered negative for both ER (n=101) and PR (n=158), as well as 16 ER+/PR+ cases that showed unfavourable outcome, by this IHC technique, were re-evaluated, whenever possible on the same paraffin blocks, using the IHC technique (peroxidase-indirect-polymer technique performed on a Ventana Benchmark *ULTRA* instrument; Ventana Medical Systems, Inc., Tucson, USA) currently employed in our laboratory. The results were recorded semiquantitatively as the percentage of positively stained neoplastic cell nuclei using ≥ 1% cut-off value as criterion for positivity (Hammond et al. 2010). For prognostic purposes, a complementary data analysis was further performed, using a >10% cut-off point. The intensity of staining was not evaluated.

### Statistical analysis

The comparative analysis of matched-paired cases for hormone receptor expression between the two IHC methods was assessed by the McNemar’s test with continuity correction. The associations of ER and PR status with clinico-pathological characteristics and outcome events (disease recurrence and death from the disease) were evaluated by Pearson’s Chi-Squared test. The probability of survival was estimated by means of the Kaplan-Meier method, and survival curves compared using the log rank test. P values < 5% were considered statistically significant.

## Results

By using the IHC method of the 90’s, 259 (71.9%) and 202 (56.1%) of 360 cases were considered positive for ER and PR, respectively. Specifically, the hormone receptor expression was as follows: ER+/PR+ (n = 196), ER-/PR- (n=95), ER+/PR- (n = 63), and ER-/PR+ (n = 6).

**Figure 1:**
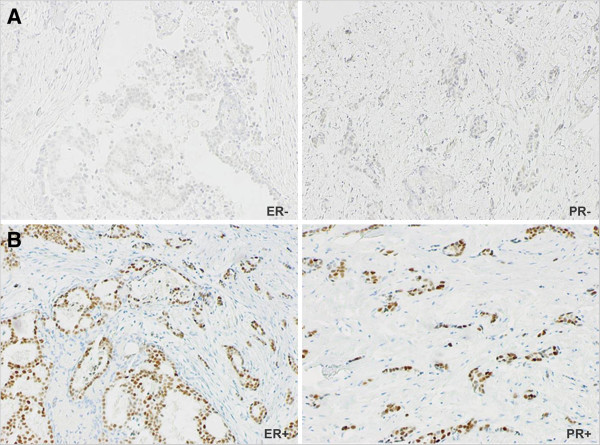
**Immunohistochemical analysis (x10) of hormonal receptors in a breast cancer case A) Previously considered double-negative and B) Its change to ER/PR double-positivity by the current IHC method.**

All cases negative for ER (n = 101) and PR (n = 158), as well as 16 ER+/PR+ cases that showed unfavourable clinical outcome, were re-evaluated using the current IHC method. The specific changes in the hormone receptor status, as compared to the previous IHC technique, were as follows: 20 (21.1%) and 12 (12.6%) of 95 ER-/PR- cases were classified as ER+/PR+ (Figure 1) and ER+/PR-, respectively; also, 38 (60.3%) of 63 previous ER+/PR- cases became PR positive. Of noting, all 6 ER-/PR+ cases changed to ER positive. All 16 ER+/PR+ cases, used as control for positive expression, maintained their co-positivity, and therefore, the observation was extended to the remaining ER and PR positive cases. Overall, using the currently recommended ≥1% cut-off (Hammond et al. 2010), 297 (82.5%) cases were considered ER positive, while PR positivity was found in 260 (72.2%). Specifically, the hormone receptor status assessed by the current IHC method was as follows: ER+/PR+ (n = 260), ER-/PR- (n = 63), and ER+/PR- (n=37).

The comparison between the two IHC techniques showed significant differences in hormone receptor expression (P<0.001; McNemar’s test), even when a >10% cut-off was applied for the current IHC method (P<0.001). Using this IHC method, 38 of 101 (37.6%) and 58 of 158 (36.7%) previously considered ER- and PR- cases, respectively, changed to positive. Of these, 4 of the new 38 ER+ (10.5%) and 21 of 58 PR+ (36.2%) cases presented a low positivity staining, i.e., between the two cut-off values used, 1% and 10%.

Table 3 summarizes the associations of hormone receptor expression with clinico-pathological characteristics, DNA ploidy and outcome measures using both IHC methods and different cut-off values for defining positivity. In general, there were no differences between them in relation to the former parameters, with the lack of ER and PR being associated with DNA aneuploidy and tumours with greater size and higher grade of differentiation. No significant association was observed in relation to axillary lymph node involvement as well.

**Table 3:** **Associations of hormone receptor expression with clinico-pathological features, DNA ploidy and outcome measures using two IHC methods and different cut-off values for positivity**

Variables	ER expression			PR expression		
	(P values)			(P values)		
	Previous method	Current method	Current method	Previous method	Current method	Current method
	(>10%)	(≥1%)	(>10%)	(>10%)	(≥1%)	(>10%)
Grade of differentiation						
G1 *vs*. G2+G3	<0.001	<0.001	<0.001	<0.001	<0.001	<0.001
Tumour size						
pT1 *vs*. pT2+pT3	0.017	<0.001	<0.001	0.003	<0.001	<0.001
Nodal status						
pN0 *vs*. pN1	NS	NS	NS	NS	NS	NS
DNA ploidy						
Diploid *vs*. Aneuploid	0.001	<0.001	<0.001	0.021	<0.001	0.002
Disease recurrence						
No *vs*. Yes	NS	NS	NS	0.032	NS	0.008
Death from the disease						
No *vs*. Yes	NS	NS	NS	0.010	NS	<0.001

*ER* Estrogen receptors, *PR* Progesterone receptors.

During follow-up time (median, 124.5 months; range, 1–240), 124 patients (34.4%) experienced disease recurrence, while 92 patients (25.6%) died from the disease. Significant associations between negative PR expression and the outcome events were observed either with the IHC method of the 90’s (although weak) or with the current one, but only when a > 10% cut-off value was used (Table 3).

**Figure 2:**
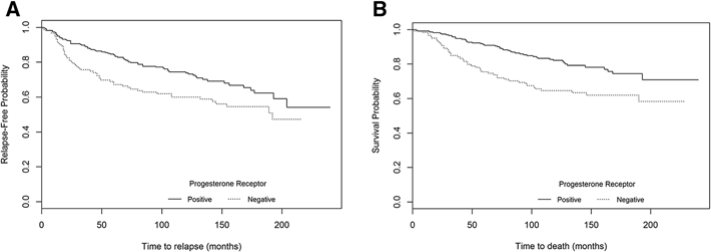
**Probability of patients' survival in the whole series (n=360) according to PR expression using the current method with >10% cut-off A) RFS (P=0.010) and B) OS (P<0.001).**

**Figure 3:**
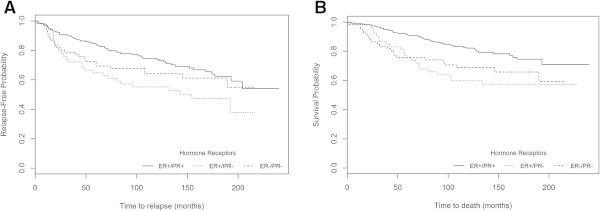
**Probability of survival in the whole series (n=360) according to ER/PR subsets of patients using the current method with >10% cut-off A) RFS (P=0.013) and B) OS (P=0.002).**

The Kaplan-Meier survival estimates analyses showed significant differences between overall survival curves for PR expression, using the IHC method of the 90’s (P = 0.017) and the current IHC method (with a >10% cut-off) (P<0.001), the latter also showing significance in relation to disease recurrence (P = 0.010) (Figure 2). Using this method (with a >10% cut-off), we found that the subgroup of patients with ER+/PR- tumours presented the worst prognosis for RFS (P = 0.013) and OS (P=0.002) (Figure 3). Comparing the K-M curves for OS, the adverse clinical outcome in this subset of patients is more pronounced after five years of follow-up.

**Figure 4:**
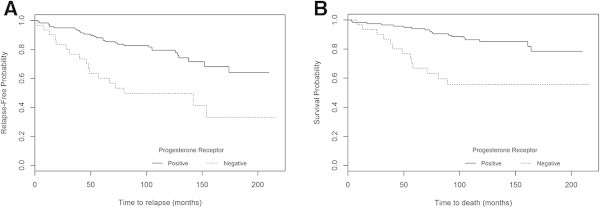
**Probability of survival in the subgroup of patients submitted to hormonal therapy (n=151) according to PR expression using the current method with >10% cut-off A) RFS (P<0.001) and B) OS (P<0.001).**

When a complementary subset analysis was performed, including only the breast cancer patients (n=151) submitted to hormonal therapy, significant differences between both the RFS and OS curves for PR were found by the current IHC method, either using a ≥1% cut-off (P=0.015 and P=0.020, respectively) or a >10% cut-off (P<0.001 and P<0.001, respectively) (Figure 4).

No statistically significant differences were observed for any ER K-M survival curves.

## Discussion

Lack of intra- and inter-laboratory reproducibility of hormone receptor expression can affect the clinical usefulness of the biomarkers as predictors of the response to endocrine therapy in breast cancer (Rhodes et al. 2001; Regitnig et al. 2002). It is, therefore, a clinical priority any attempt made for improving the accuracy of the IHC technique. In this light, our study sought to investigate potential differences of hormone receptor expression between two IHC methods separated in time, by re-evaluating, on the same tumour material, ER and PR analyses performed in the 90’s.

All cases previously considered ER and PR negative, together with 16 of 196 ER+/PR+ cases, were re-analysed by using a current IHC method. The latter 16 cases were selected, throughout the decade, for having exhibited adverse clinical outcome, and served as control for testing ER and PR positive expression. It seemed to us reasonable to think that these cases would not change their hormone receptor expression, due to the higher cut-off value used in the IHC method of the 90’s. The hypothesis was further confirmed, as all 16 cases maintained their ER/PR co-positivity, and inferred for the remaining ER and PR positive tumours.

The matched-paired McNemar’s test revealed significant differences of hormone receptor expression between the two IHC methods. The data showed that a substantial proportion of previous ER (37.6%) and PR (36.7%) negative cases changed to positive. In consequence, ER and PR positivity increased from 71.9% and 56.1% to 82.5% and 72.2%, respectively. These changes do not appear to be caused only by the distinct cut-off values used for both methods, since when an identical cut-off point (>10%) for the current IHC method was applied, significant differences remained. Instead, the fact could be better explained by the IHC technical evolution over time, through automated procedures that allow a superior level of standardization as compared with previous manual staining methods. Different primary antibodies and improved detection systems could be the main causes involved.

In our study, as reported by others (Collins et al. 2005; Khoshnoud et al. 2011), the vast majority of breast carcinomas showed essentially a bimodal distribution of ER staining, varying between diffusely positive or completely negative ER expression. Indeed, we observed that only 4 of the new 38 ER+ cases had low positive nuclear staining, i.e., ranging between 1% and 10%. Interestingly, these cases were associated with lack of PR expression and poor prognosis (one recurrence and two deaths from the disease; data not shown). The rarity of the finding, which some authors attributed to inadequate fixation or focal tumour necrosis (Nadji et al. 2005), suggests that ER quantification may be, in practice, unnecessary or superfluous. Welsh et al. (2011) showed that changing the percentage of positive cells from 10% to 1% cut-off, as recommended by the new American Society of Clinical Oncology/College of American Pathologists (ASCO/CAP) guidelines (Hammond et al. 2010), did not affect significantly the overall number of ER-positive patients. However, our data point out that a special attention must be focused on these individual cases that, although unusual, tend to have an unfavourable clinical evolution and might benefit from adjuvant chemotherapy. In addition, it should be noted that all six previously labelled ER-/PR+ breast carcinomas changed to ER positive, which strongly suggests that this putative subset may represent a mere technical artifact (Rakha et al. 2010). Accordingly, Nadji et al. (Nadji et al. 2005), in their large immunohistochemical study of 5,993 breast cancers, found no ER-/PR+ tumours.

On the other hand, the assessment of PR immunostaining revealed prognostic significance, especially when using a >10% cut-off value. This suggests that, with regard to clinical outcome, cases with a low positive PR level (between 1% and 10%) do not differ significantly from those considered as PR negative. Together with the few ER low positive tumours, the finding raises the clinical question of whether patients with low positive hormone receptor expression would actually benefit from endocrine treatment. Our data seem to indicate that the pros (therapeutic benefit) and cons (potential side effects) of giving hormonal therapies for patients with minimal ER and PR positive expression should be cautiously evaluated. In a recent study, aiming to investigate the impact of low ER and PR expression (<10%) as well as the effect of endocrine therapy on survival outcomes of 1,257 previously classified triple negative breast cancer patients, Raghav et al. (Raghav et al. 2012) observed that for both ER/PR 1%-5% and 6%-10% level subgroups, no prognostic utility and only a tendency for survival advantages were found, respectively. These controversial findings reveal that the application of endocrine therapy in these patients needs further investigation (Brouckaert et al. 2013).

As expected, our data showed the significant association of negative hormone receptor expression with DNA aneuploidy and adverse clinico-pathological features, such as greater size and higher grade of differentiation tumours. In keeping to others (Jalava et al. 2005), no correlation was found between ER or PR and axillary lymph node status, suggesting that the biomarkers are not predictors of metastatic potential. However, only the lack of PR expression was associated with disease recurrence and mortality, using both the IHC method of the 90’s and the current one with a >10% cut-off value.

The Kaplan-Meier survival curves estimates did not show the prognostic significance of ER expression by any IHC method. On the contrary, it was proved the significant prognostic impact of PR analysis either for relapse-free or disease-specific survival of patients with breast invasive ductal carcinoma. The same applies when a complementary subset analysis was performed in the subgroup of patients submitted to hormonal therapy. Interestingly, the prognostic significance of PR expression was strongly evident (lowest P value) when using a >10% cut-off point for the current IHC method, highlighting the importance of selecting this cut-off in the assessment of tumours positivity for better discriminating patients into two groups with distinct survival. Ogawa Y et al. (Ogawa et al. 2004), in their immunohistochemical study of 249 female breast cancers, reached the highest prognostic impact when they adopted an identical cut-off point (>10%) for hormone receptors in patients treated with endocrine therapy.

It was very striking, using the current IHC method with a >10% cut-off value, the worst prognosis found in the subgroup of patients who presented ER+/PR- tumours. As reported by others (Viale et al. 2007; Thakkar & Mehta 2011; Arpino et al. 2005), the latter seem to be a distinct subset of breast carcinomas characterized by great genomic instability, high proliferation rate, and aggressive behaviour, being associated by gene signature with the luminal B subtype (Perou et al. 2000). ER+/PR- tumours would represent, at a molecular level, a different subtype, as compared with ER+/PR+ and ER-/PR- breast carcinomas (Creighton et al. 2009). Although the biological role of PR is not yet fully elucidated, the PR downregulation might be an indicator of a nonfunctional nuclear ER pathway or the (gene silencing) result of the *PR* promoter methylation (Cui et al. 2005). In the clinical setting, the lack of PR in ER+ tumours could be predictive of poor response to endocrine therapies (Bardou et al. 2003; Rakha et al. 2007).

In conclusion, the present data indicate that the usefulness of automated methods, as well as more specific and sensitive detection systems, has significantly contributed to improve IHC techniques for determination of hormonal receptors in breast cancer. In particular, the comparison of ER and PR analyses performed in the 90’s and nowadays, emphasizes the clinical relevance of the reappraisal of negative hormone receptor expression in the former, owing to the decrease of false negative results. Furthermore, it was confirmed the prognostic significance of PR status, mainly when using a >10% cut-off value, either in the whole series or in the subgroup of patients who received hormonal therapy. Finally, it should be highlighted the fact that patients who presented ER+/PR- tumours exhibited the worst prognosis, which could have therapeutic implications in the management of breast cancer disease.

## Ethical standards

The experiments comply with the current laws of the country (Portugal) in which they were performed.
